# Developing Language and Image Use Guidance for Communicating About Suicide, Mental Health Concerns and Alcohol and Other Drug Concerns: An Australian Delphi Study

**DOI:** 10.1002/hpja.70236

**Published:** 2026-08-14

**Authors:** Elizabeth Paton, Jennifer Peprah, Dara Sampson, Jaelea Skehan

**Affiliations:** ^1^ College of Human and Social Futures, University of Newcastle Callaghan Australia; ^2^ Everymind Newcastle Australia; ^3^ College of Health, Medicine and Wellbeing, University of Newcastle Callaghan Australia

**Keywords:** alcohol and other drugs, best practice communication, guidelines, mental health, suicide

## Abstract

**Issue Addressed:**

How people communicate about suicide, mental health concerns and alcohol and other drug (AOD) concerns matters. The words and images used can reinforce or reduce shame and stigma, or impact help‐seeking and help‐offering behaviours. In some cases, how people communicate may increase or decrease suicidal behaviour in Australia communities.

**Methods:**

This paper outlines findings from an Australian Delphi survey to find consensus on what constitutes best practice advice for communicating about mental health concerns, suicide, and AOD concerns. Three panels, including a panel of people with lived experience, were used by the researchers to achieve consensus amongst those considered experts where evidence and consistency was lacking.

**Results:**

Consensus ratings and qualitative feedback were received from 40 experts across two Delphi survey rounds. In total, 70 of 106 statements were endorsed for inclusion in guidelines for image or language use relating to mental health, mental health concerns, suicide and AOD concerns.

**Conclusions:**

Consensus ratings and qualitative feedback provided clear, broad‐level guidance that formed the foundation for two new sets of guidelines. This builds on existing language focused guidelines to deliver updated and evidence‐informed guidelines for use in the media as well as other sites of communication where people may be exposed to harmful or helpful words and images.

**So What?:**

These new guidelines are designed to help Australian communities to communicate safely and sensitively, and with a more coherent voice, about suicide, mental health concerns and AOD concerns. In turn, they contribute to broader efforts to reduce stigma and distress across Australian communities.

## 1 Introduction

When it comes to mental health concerns, suicide or alcohol and other drug (AOD) concerns, media and other professional communicators, such as podcasters, content creators or communications staff embedded in sector organisations or government agencies, informing communities and engaging them in conversations about these issues is critical for prevention, intervention and postvention efforts as well as broader policy and strategy development. *What* and *how* they communicate also matters. Multiple studies have shown, for example, the breadth of effects associated with media portrayal and other public communication about these issues. The language and images used can contribute to harm within the community, such as increasing rates of suicidal behaviour, stigma and discrimination [1, 2, 3, 4]. Conversely, they can help reduce fear and shame, increase knowledge, and promote increased help‐seeking and help‐offering behaviours, ultimately lowering the risk of suicide [5, 6, 7, 8].

### 1.1 Communication Guidelines

Based on this research exploring the links between public communication and its impact on communities, health, mental health and suicide prevention organisations and media agencies developed guidelines for communicating about suicide, mental health concerns and AOD concerns. Internationally, this guidance is predominantly focused on media reporting, given the broader scope for community impact than other forms of communication. This includes guidelines by the World Health Organization [9] as well as guidelines in countries such as Australia [10, 11], New Zealand [12, 13], Hong Kong [14], Malaysia [15], Nigeria [16], the United Kingdom [17] and the United States [18] amongst others. More recently, as increased evidence has emerged, further guidance was developed to complement these media guidelines and expand into other forms of public communication. The #chatsafe guidelines, for example, were developed by Australian youth mental health organisation Orygen to support young people communicating about suicide on social media [19, 20].

Recommendations for safe and sensitive media reporting are largely consistent across the guidelines, with all advocating against inclusion of harmful material, such as details of suicide methods, and for inclusion of prevention‐focused story elements, such as details of helplines and other supports as well as stories of survival, hope or recovery [3]. Newer or updated guidelines also included considerations for how digital environments, such as streaming services or social media, may impact application of these recommendations [9, 20].

Implementation of media guidelines, particularly in the Australian context through the Mindframe program, has been shown to be impactful and cost‐effective prevention activities [21, 22, 23]. Studies on the effectiveness of media guidelines and their implementation showed changes in journalist attitudes and behaviours, and an association with reduced suicide rates [22]. A more recent study comparing implementation of guidelines in regions of Spain and Australia showed simple or one‐off interventions (such as training) could improve adherence to recommendations but that longer‐term or ongoing interventions (such as those provided through the Mindframe program with regular and repeated training in newsrooms and university settings, real‐time support for journalists and inclusion in codes of practice and editorial standards) were more effective [21].

### 1.2 Guidelines for Broader Language and Image Use

Despite the considerable support available in Australia around public communication of suicide, mental health concerns and AOD as outlined above, the national conversation about mental health concerns, suicide and AOD concerns continues to grow and there is need for more coherent guidance on the use of language and images across communication mediums. To further enhance Australian guidance on safe and effective use of language and images, Everymind, a national mental health and suicide prevention institute that implements the Mindframe program, was funded by the National Mental Health Commission to review the evidence, consult with stakeholders and develop and disseminate new guidelines for the Australian context (the Words and Images project).

The project commenced with a lived experience roundtable discussion to shape the project direction. Some of the participants from the lived experience round table were also invited to join a project advisory group to provide continual support and insight into the project development. The remaining members of the project advisory group included prominent figures in the Australian mental health and wellbeing, suicide prevention and AOD sectors.

Throughout the scoping phase, Everymind engaged in a systematic scoping review of the existing literature on image use, a scoping review on the existing guidelines on language and image use, a consultation survey on image use and stigma, a scoping review of language used by governments and the suicide prevention sector in Australia, and focus group discussions.

The lived experience roundtable brought together people with a diverse range of lived and living experience to support and define project research questions and approaches to creating a set of guidelines. Discussion identified that current use of images is not representative of what it is like to experience a mental illness or suicidal distress. Representatives highlighted the importance of images and words to help reduce stigma and increase public knowledge and acceptance of people living with mental health concerns, AOD issues or suicidality. During the lived experience roundtable event there were different, and sometimes contradictory, perspectives presented, which reinforced the need to consider the complexity of portraying these issues through language and images, emphasise choice and to acknowledge the evolving and personal nature of language.

The systematic scoping review of research focused on image use found that use of images by a range of stakeholder groups including media had the potential to impact how people with lived experience of mental health concerns, suicide or AOD use were perceived at a population level. Findings from review of 40 studies (38 peer‐reviewed, 2 grey literature) indicated that images chosen in public communication were often sensationalised and stigmatising. Across media platforms, for example, people with mental health concerns were commonly presented as dangerous, objects of ridicule or unlikeable. Despite this, evidence on best‐practice use of images as well as what types of images may be preferred by people with a lived experience was lacking. A consultation survey conducted by Everymind on image use by media, sector professionals and people with lived experience found a similar focus on safety/avoiding harm (40%) and avoiding stigma in image use (33%) over a focus on positive or hopeful images (18%). The consultation survey also revealed that organisations were most commonly using images in public facing communications, including marketing/promotional work, websites and social media. These images were most often sourced from an external stock image database, and selected by the communications, branding or media teams.

A scoping review of guidelines for language and image use currently used by organisations analysed 43 sets of guidelines (supplied by 88 sector organisations, Primary Health Networks, government departments, media and social media organisations). The information provided in the guidelines was mostly consistent, with many specifically referencing or paraphrasing the Mindframe guidelines. Most guidelines provided general advice or were language specific, with only 10 including guidance on image use. Guidance was largely focused on safety and what to avoid, with a smaller number providing alternatives or what‐to‐do advice. The most significant difference between the guides was the depth and specificity of information included, as well as a noticeable difference in the style of writing, varying between authoritative ‘rules’, or more relaxed and limited in providing clear instruction on language and image use.

A scoping review of language used by governments and the suicide prevention sector in Australia analysed glossaries or key definitions included in government policies and strategies related to mental health and suicide prevention as well as language used on websites for organisations in the mental health and suicide prevention sectors. When comparing key terms defined via the glossary review process against web‐based language within the sector, the lack of universal usage of language was a recurrent theme; however some emerging consensus was found about broad terminology. Use of the term ‘mental health’ to refer to a positive state of mental wellbeing was consistent, for example, across most glossaries and websites. Emerging consensus was also found for the absence of mental health (‘mental health issues’, ‘mental ill health’, ‘mental health problems’), for the absence of mental health and clinical symptoms/diagnoses (‘mental illness’, ‘mental health disorders’) and for suicidal behaviour (‘suicidal behaviour’). While this consensus exists on several concepts in national level policies, strategies or websites, a lack of consensus remains on more specific terminology, including the use of culturally specific language. This is consistent with other research that shows studies have yet to meaningfully interrogate the cultural specificity of language beyond language deficit or disparity approach, and whether a common language could promote understanding and challenge public levels of stigma [24].

### 1.3 Preliminary Work

A series of focus groups was conducted to further explore what key stakeholders (including people with lived experience) saw as the key issues and priorities to be addressed in the language and image guidelines [25, 26]. A total of 49 participants took part across 10 sessions, from the perspective of professional communicators, people with professional or personal experience of suicide, mental health concerns or AOD, or who work within the MHSP sector, and people identifying as or working with priority populations (including men, young people, LGBTIQ+ people, people from culturally and linguistically diverse backgrounds [CALD] and Aboriginal and Torres Strait Islander people). Across focus groups, participants indicated that safe language and image use required ongoing engagement with the principle of ‘do no harm’ rather than prescriptive or static rules. While it was noted that this was a complex and challenging task, participants shared broad and important insights into what this approach might look like. Four overarching themes emerged from analysis of the transcripts including the importance of story and narrative; that diversity is needed, but there are challenges; that guidance should be context specific, rather than generic; and there should be a sense of balance, community, and connection.

These activities provided the background and foundation of the current study, where Everymind explored whether there was consensus around what constitutes best‐practice use of images and language. The study aimed to develop and refine guidance to support individual and organisational communicators such as communicators in the media, suicide prevention, mental health and AOD sectors, government departments, and the advertising/fundraising industries across Australia in using language and images related to suicide, mental health concerns and AOD concerns. To achieve the stated aim, the study sought to answer the following research questions:

1. What level of expert consensus can be achieved on proposed guidelines statements about the use of images relating to mental health, mental illness, suicidal behaviour and alcohol and other drug use?
2. What level of expert consensus can be achieved on proposed guidelines statements about the use of language relating to mental health, mental illness, suicidal behaviour and alcohol and other drug use?

## 2 Methodology

The study was conducted by Everymind, in conjunction with researchers from The University of Newcastle. The study used a two‐step modified Delphi method [27], consisting of qualitative assessment and a ranking evaluation (‘administration’), and consensus meetings between researchers. The Delphi method was used to establish levels of consensus between a wide range of stakeholders, such as media and communications professionals, people with a lived experience and people working with various community groups in the mental health and suicide prevention sectors.

Ethical approval for this study was provided by NSW Hunter New England Local Health District Research Ethics Committee (HNELHD‐HREC, 2022ETH00314).

### 2.1 Delphi Process

**FIGURE 1:**
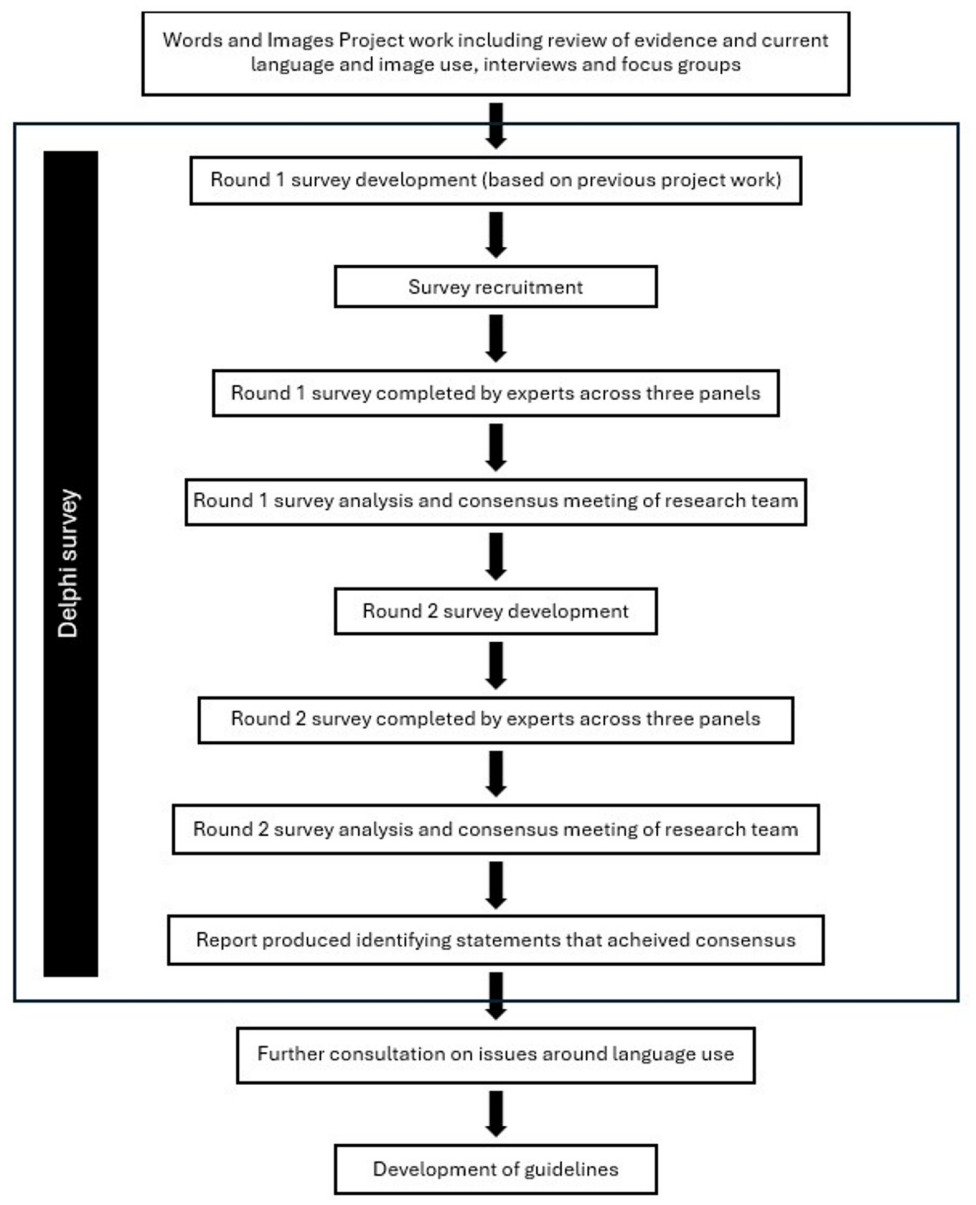
Delphi process.

The Delphi method is a consensus‐based research technique that provides a systematic means of collecting and aggregating the judgements of a group of experts via multiple iterations (also known as ‘rounds’). When evidence is lacking, this process recognises the value of expert experience and opinion [28], with shared feedback from each sequential round allowing each expert participant to reconfirm, reassess and/or further develop their position. It also provides an opportunity to address traditional power structures within research, with space to determine what counts as expertise and who counts as an expert. In this case, people with a lived and living experience of suicide, mental health concerns and AOD concerns were given equal footing with researchers and those working in health and media sectors. While not all experts identified as Australian, all experts were based in Australia at the time of the study. The Delphi method has been applied successfully in health research generally, and in the development of key messages and guidelines more specifically [19, 20, 29, 30]. This included the development of Australian guidelines for young people communicating about suicide online [19, 20] and for media reporting on severe mental illness in the context of violence and crime [31]. Other media, language or image guidelines were developed using other stakeholder consultation methods such as interviews, focus groups, non‐Delphi surveys and review of pre‐drafted material [9, 11, 12, 13, 14, 15, 16, 17]. To the best of our knowledge, this study represented the first application of the Delphi technique, in Australia or internationally, to specifically focus on safe use of words and images in public communication about mental health, mental health concerns, suicide and AOD concerns. See Figure 1 for an overview of the Delphi process explained below.

### 2.2 Survey Development

Initially, a comprehensive list of statements was developed using evidence from the wider Words and Images project as detailed earlier. This included: (i) results of scoping reviews of academic evidence; (ii) results of scoping reviews of existing guidelines and policies; (iii) scoping reviews of current language use across government, mental health, suicide prevention and AOD organisations in Australia; (iv) a national consultation survey on image use and stigma; and (v) consultation via focus groups [26]. The statements were initially drafted by a social work researcher at the University of Newcastle familiar with the evidence, reviewed by research and project staff at Everymind, and finalised during a collaborative workshop. The workshop provided an opportunity for researchers to review and compare draft guideline statements, reflect on the existing evidence, and engage in a collaborative development process. Final statements were grouped into subcategories during the workshop to help streamline the rating process. The Round 1 survey included 99 items for rating (43 language statements and 56 image statements). Example statements include:

- Language should not present suicide as a desired outcome (e.g., ‘successful suicide’ or ‘unsuccessful attempt’).
- It is confusing when people use different terms to mean the same thing in mental health (e.g., ‘mental health difficulties’, ‘mental ill‐health’, ‘mental health challenges’).
- Images that show people as threatening, aggressive or violent can reinforce harmful stereotypes and should be avoided.
- Images used to communicate about people with alcohol and other drug problems should not show ‘before’ and ‘after’ photographs of a person.

### 2.3 Sampling and Recruitment

The research team recruited participants (‘experts’) to form three expert panels, which consisted of:

- Panel A—Professional communicators (including media)
- Panel B—Sector professionals from the mental health and suicide prevention field
- Panel C—People with personal experience of suicidal behaviour, mental health concerns, and/or alcohol and other drugs

Experts were eligible to participate if they were: (i) aged 18 years or older; (ii) currently living in Australia; (iii) able to undertake the survey in English; (iv) able to be allocated to one of the above panels based on their work as a professional communicator or in the mental health, suicide prevention or AOD sectors, or as a person with a lived experience of suicide, mental health concerns or AOD concerns. A person with a lived experience was defined as someone with direct, personal experience of suicidality or suicide attempt, mental health concerns or alcohol and other drug use; someone with personal experience of caring for someone through a crisis, illness, episode or use; and/or someone with personal experience of suicide bereavement.

Recruitment was both purposive and non‐probability convenience sampling, managed by Everymind to invite experts based on expertise relevant to the study and using known networks of expertise. These networks included: advisory groups and contact lists for Australian media and other professional communicators; lived experience networks; and the mental health, suicide prevention or AOD sectors, including organisations focused on priority populations such as young people, LGBTIQ+ people, Aboriginal and Torres Strait Islander people, people from a culturally and linguistically diverse background, and men.

### 2.4 Data Collection and Analysis

Experts received a link to the first‐round online survey (using the REDCap system), where a participant information statement provided a rationale for the study and instructions for completing the survey. Informed consent was provided in the survey. A link to the second‐round survey was emailed following analysis. Experts had approximately 2 weeks to complete each survey round.

In the first round, each expert was asked to answer demographic questions and then rank each of the 99 statement items according to their importance for inclusion in a set of guidelines related to safe, inclusive, and non‐stigmatising communication about mental health and wellbeing, mental illness, suicidal behaviour, and AOD concerns. Rating occurred on a five‐point Likert scale that included the following options: *essential, important, unsure/depends, unimportant, should not be included*. There was also an option for a qualitative comment or suggestion after each item to be rated.

Basic statistics were generated by the REDCap survey software. Excel software was used to map which statements achieved consensus. Consensus ratings were developed for each panel by calculating the percentage of experts rating the statement as ‘important’ or ‘essential’. Statements were eligible for inclusion in the round two survey and final guideline statements if they aligned with the modified classification system of the study. This approach to rating aligns with previous studies [29, 30, 31, 32], where responses to individual statements were classified based on simple rules:

**Inclusion:**

1. Attained > 80% consensus in each of the expert panels.
2. Attained > 70% consensus in each of the expert panels and qualitative responses OR evidence indicated that it was to be included with modification.

**Re‐rate:**

3 Attained > 50% and < 70% consensus.
4 Attained < 50% consensus, however qualitative responses indicated confusion about the statement meaning and thus called for modification and re‐rating.

**Exclusion:**

5 Attained < 50% consensus.

Trends in ratings between the three panels were analysed more broadly by comparing and contrasting consensus rates. Qualitative comments were summarised by the researchers and a thematic analysis applied.

The results of the first round were tabulated and analysed using the rating system described above. Some statements related to specific language and terminology were excluded from the Round 2 survey due to qualitative responses and consensus levels indicating a need for further consultation outside of the Delphi process. All remaining statements, including those with divergent consensus or confusion indicated in the qualitative responses, were modified for clarification and included in the second survey to be re‐rated. Finally, researchers analysed the qualitative responses for feedback that constituted a new idea. These were written up as new statements for inclusion in the second‐round survey.

The second‐round survey included 37 statements for re‐rating and seven new statements. Experts were also provided with summary statistics from the first round. Following the second Delphi round analysis, researchers held consensus meetings to assess all available evidence for language and images and to consider statements to be included considering expert comments, summary statistics, and the wider research evidence.

## 3 Results

### 3.1 Participants

Of the total 40 experts who completed the first round, the majority identified as women or female (*N* = 32, 80%). Experts ranged in age from 18–24 (*N* = 2) to 65–74 (*N* = 3), with the highest proportion aged 35–44 (*N* = 11, 27.5%) and 45–54 (*N* = 11, 27.5%). Two experts identified as Aboriginal or Torres Strait Islander (5%). Four experts identified as culturally and linguistically diverse (10%), and 10 were born in a country other than Australia (25%), predominantly the UK (*N* = 5, 8%). Nine experts indicated a sexuality other than straight or heterosexual (22.5%). Finally, 11 experts (27.5%) identified themselves as ‘Professional communicators (including media)’; 19 (47.5%) identified themselves as ‘Sector professionals from the mental health and suicide prevention field’; and 10 (25%) indicated ‘Personal experience of suicidal behaviour, mental ill‐health or alcohol and other drug concerns’. These role/professional demographics were used to assign experts to one of the three expert panels. Experts assigned to the lived experience panel further identified their experiences of mental health concerns, suicide and AOD concerns (see Table 1 for full participant demographics).

**TABLE 1:** Demographics of participating experts.

Demographics	No.	%
Current age		
18–24	2	5%
25–34	8	20%
35–44	11	27.5%
45–54	11	27.5%
55–64	5	12.5%
65–74	3	7.5%
75+	0	0
Gender		
Man or male	6	15%
Woman or female	32	80%
Non‐binary	0	0
I use a different term	1	2.5%
Prefer not to say	1	2.5%
Sexual orientation		
Straight (heterosexual)	30	75%
Gay or Lesbian (homosexual)	3	7.5%
Bisexual or pansexual	3	7.5%
Asexual	1	2.5%
Queer	0	0
I use a different term	2	5%
Prefer not to answer	1	2.5%
Aboriginal or Torres Strait Islander Origin		
Aboriginal	2	5%
Torres Strait Islander	0	0
Aboriginal and Torres Strait Islander	0	0
Neither Aboriginal nor Torres Strait Islander	37	92.5%
Prefer not to say	1	2.5%
Identifies as culturally and linguistically diverse		
Yes	4	10%
No	35	87.5%
Prefer not to answer	1	2.5%
Country of birth		
Australia	30	75%
Country other than Australia	10	25%
UK/England	5	12.5%
New Zealand	2	5%
Saudi Arabia	1	2.5%
Sweden	1	2.5%
Netherlands	1	2.5%
Type of lived experience (lived experience panel experts only; multiple responses allowed)		
Mental ill‐health, mental illness or psychological distress	7	70% (of 10)
Alcohol and other drug concerns	3	30% (of 10)
Suicidal distress	6	60% (of 10)
Poor social and emotional wellbeing	4	40% (of 10)
Caring for someone in suicidal distress	3	30% (of 10)
Caring for someone with a mental illness or psychological distress	7	70% (of 10)
Caring for someone with alcohol or other drug concerns	3	30% (of 10)
Bereavement by suicide	2	20% (of 10)

### 3.2 Panel Retention

Of the total of 40 experts who completed the first round, 25 completed the second round. Retention was highest in the professional communicators (including media) panel (9/11; 81.8%), followed by the lived experience panel (7/10; 70%). Poor retention in the sector professionals panel (9/19; 47.4%) dropped the overall retention rate to 62.5% (25/40).

### 3.3 Round 1 Survey

In the first round, 23 of 56 statements (41%) related to image use and 31 of 43 statements (72%) related to language use achieved consensus across all three expert panels. A total of 13 statements were excluded, with the remaining 32 statements indicating some consensus (mid‐range consensus across all three panels or consensus across two of the three panels) or the need to alter the statement and re‐rate (see Table 2). The preliminary analysis figures were subject to change following further analysis of the qualitative feedback discussed below.

**TABLE 2:** Round one analysis.

	Images	Language	Total
Re‐rate	22	10	32
Exclude	11	2	13
Include	23	31	54
Total	56	43	99

Round 1 qualitative findings reflected the overarching complexity of the project, especially with regard to language. Qualitative feedback indicated further wordsmithing of statements was required including for several that had met criteria in the first round for either inclusion or exclusion as well as some where consensus was not reached. These items were reworded based on this qualitative feedback and included in the second survey for re‐rating based on the new wording.

Qualitative feedback also reinforced the subjective nature of language and indicated that further consultation was needed with regards to statements that recommended particular words or phrases be used or avoided. For example, although the statement achieved consensus, several experts indicated different opinions on avoiding terms that indicate a lack of quality of life such as ‘victim’ or ‘suffering from’:

- ‘Since when does the term ‘suffering’ suggest a lack of quality of life? It is an experience and a truth. The term victim however is very cliche and should be avoided’.
- ‘I'm a bit on the fence with this one. I think it's good to take the empowered approach to discourage people from using this language, but in my experience some people do strongly identify with these terms in the context of their own experiences. I do think we want to be using empowering terms where possible though, like the example above about “lives with” “manages” etc.’
- ‘It is best to check with that group of people to see what terms they like’.

A decision was made not to progress three terminology specific statements to round 2, although they met criteria for re‐rating, with a recommendation for further consultation, particularly with people who have a lived experience.

Qualitative feedback received in Round 1 was also reviewed for any content that constituted an entirely new idea or concept. These were crafted into new statements and included in the second round of the survey only.

### 3.4 Round 2 Survey

The Round 2 survey included a summary of the Round 1 survey consisting of descriptive statistics about the results. Experts were then asked to rate 44 items (6 language statements for re‐rating and 2 new; 31 image statements for re‐rating and 5 new), using the same five‐point Likert scale. Final responses were analysed as described for round 1, with the modification that statements that did not meet inclusion or exclusion criteria but still achieved a relatively high consensus (> 80% in two of the three panels or > 70% across three panels) were retained for post‐survey discussion or further consultation. In total, 16 statements from the second round achieved consensus across the three panels (36%), while 25 statements met the criteria for discussion (57%), and 6 statements met the criteria for exclusion (14%) (see Table 3).

**TABLE 3:** Round two analysis.

	Images	Language	Total
Exclude	6	0	6
Include	11	5	16
Total	36	8	44

A single textbox was included for feedback in the second survey. Qualitative feedback indicated that experts felt they did not possess the required expertise to answer certain items in the questionnaire but if the statements were evidence‐based, they would be inclined to support them:

- ‘There are many of the questions here which I've answered in a way to suggest I don't know because I don't know the evidence…. So what I'm trying to say is that in relation to many of these questions ‐ my expertise doesn't extend to knowing the right thing to do… and I'm hesitant to go further than what I know—through evidence—to be problematic’.

Qualitative feedback also indicated some statements required modification for a clearer understanding of the principles underlying the statement:

- ‘Some of the reasons for not including images are a bit off… There are funny wordings in the statements and while the intent may be important; they could be misunderstood. It would be useful at some point to better wordsmith the statements’.

Finally, qualitative feedback indicated a need for guideline statements to acknowledge the diversity of understanding and approaches:

- ‘We need to be mindful about how this intersects with harm minimisation approaches (and also stigmatising people who don't achieve what is perceived as a “full recovery”). People need accurate information… Also, people need information about why some websites might be harmful’.
- ‘It is important that any communication should be assessed with a cultural lens if it is to be used with different communities. Certain words/colours/images etc. may be offensive in some cultures. Seeking out cultural advice for communication tools is advised. Also consider any translation of communications should be simple English and translated by accredited translators and where possible tested with community’.

Further consultation was recommended based on this qualitative feedback for 22 statements.

### 3.5 Final Delphi Process

**FIGURE 2:**
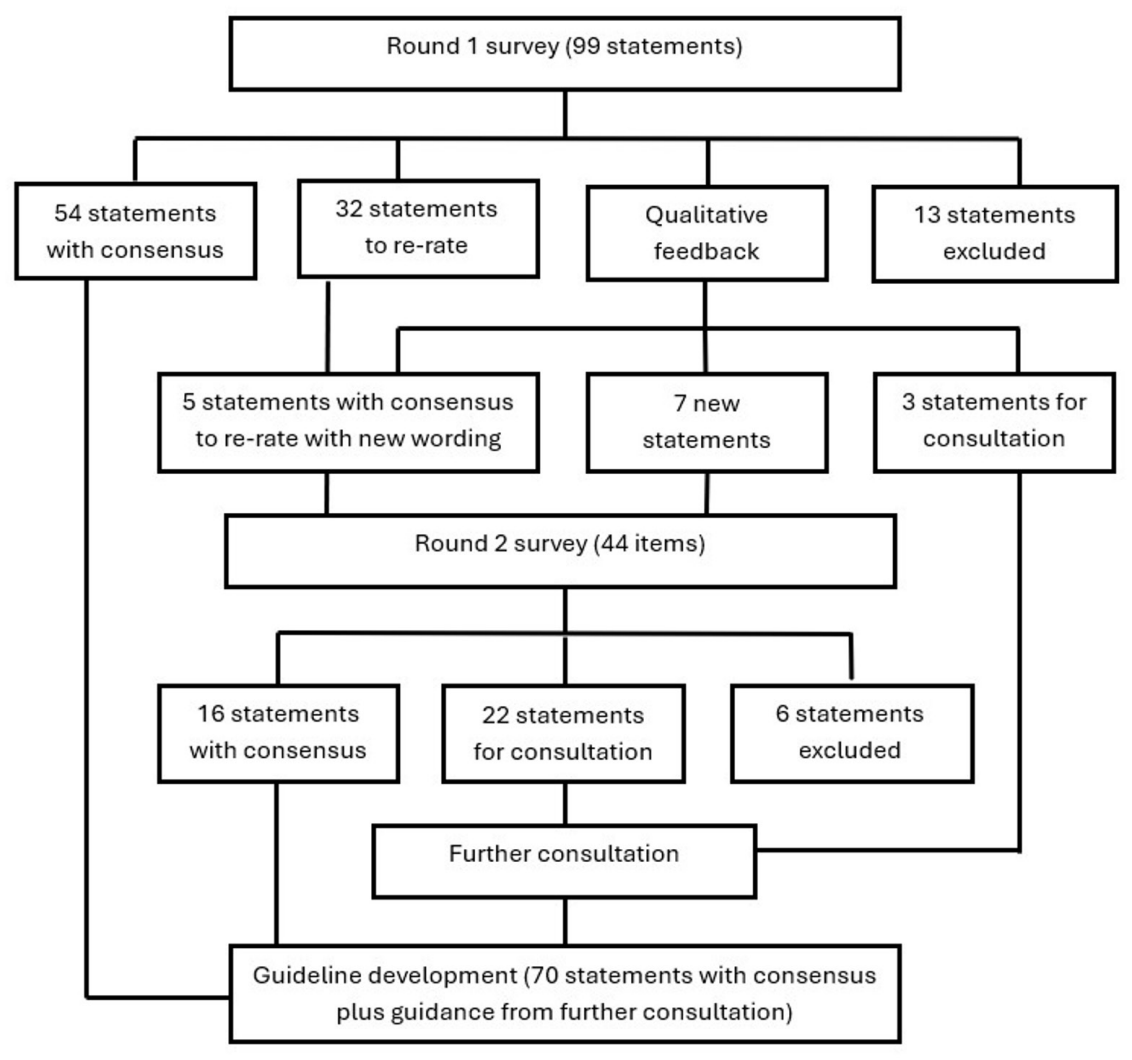
Delphi results.

Figure 2 provides a visual overview of the results. In total, 70 statements were endorsed by three panels of experts for inclusion in guidelines for image or language use relating to mental health, mental health concerns, suicide and AOD concerns (see Supporting Information Appendix A). As shown in Supporting Information Appendix A, these statements provide broad advice or what can be seen as overarching principles:

- *Language statement 1*: The language used in communication should be non‐judgemental and non‐stigmatising.
- *Language statement 11*: Person‐first language should be used when describing someone's experiences (e.g., ‘a person experiencing’ or ‘a person living with’).
- *Image statement 1*: Images should include a diversity of ages, genders, cultures and geographies.
- *Image statement 11*: Images of people who have personal or lived experience should be used with their knowledge and permission.

The statements also provide more specific advice on language or images to avoid:

- *Language statement 14*: The term ‘commit’ should not be used when communicating about suicide as it has been associated with crime and/or sin.
- *Language statement 28*: Language used to describe mental health services should not imply that they are similar to prisons (e.g., ‘discharged’ rather than ‘released’ from hospital).
- *Image statement 15*: Images should avoid depicting a power imbalance between people providing support and people receiving support.

Some endorsed statements provide more specific advice on positive or agreed image content to use.

- *Image statement 4*: Images that show people in the company of others are preferred over images that show people alone.
- *Image statement 5*: Images that show people accessing support from relevant and available services can be helpful.

Few statements on preferred definitions or the meaning of key terms achieved consensus. While some what‐to‐avoid statements provide alternate examples, the only positive or agreed terminology‐focused statements to receive consensus were on use of the term ‘mental health’.

- *Language Statement 33*: The term ‘mental health’ should be used to describe a positive concept of wellbeing, not merely the absence of mental illness.

A further 25 statements that were removed following the Round 1 survey based on qualitative feedback (3 statements) or that did not achieve consensus in the Round 2 survey but had either qualitative feedback or a significant body of evidence backing them (22 statements) were discussed at a final consensus meeting. It was determined these 25 statements required further consultation outside of the Delphi process with experts beyond those included in the three panels.

## 4 Discussion

Across the three expert panels, consensus was reached for 70 of 106 statements (99 original statements plus seven new statements generated during Round 1), reflecting general agreement across areas of expertise on overarching guidance or recommendations on language and image use. As noted earlier, the lack of agreement on preferred terminology was highlighted after the first round of the survey where qualitative feedback provided insight into the complexity regarding agreement on specific terms to be used across Australia. This complexity regarding consistent language use reflected broader debates that are inherent in any efforts to uncover or develop universally accepted language in mental health research [33, 34]. What emerged from across all elements of the Words and Images project was the evolving and largely personal nature of language; some broad language preferences change over time (as in the case of the term ‘commit suicide’), while the words people use to describe themselves and their experiences may be based on any number of individual, social or cultural factors. These findings show that there was a consensus view that consistent use of language is preferable (Language statement 35. ‘Consistent use of language relating to mental illness and suicide may help to increase understanding and decrease stigma in Australia. It is preferable that the same or similar terms are used across different sectors and communities when communicating about these issues’). However, more consultation and research were needed to understand which terms are preferred and whether there is a consensus view about the meaning of those terms. Even after these processes, it was evident that regular review of guidance on specific terms would also be necessary to capture language and related social norms as they evolve.

Overall, these findings were consistent with the recommendations found in guidelines for media reporting on suicide, mental health concerns and alcohol and other drugs [12, 19] as well as other Delphi‐derived guidelines, including for young people communicating about suicide online [19, 20] and for reporting on complex mental illness in the context of violence and crime [31]. Advice against using sensationalised language and images, for example, is present across all guidelines; where focusing on suicide, all advise against using the term ‘commit suicide’ and provide alternatives such as ‘died by suicide’.

What is notable in comparison was that this study's findings expanded on both language and image guidance provided in existing guidelines, where language or image recommendations were generally one section or one recommendation amongst other guidance. This study, for example, was unique in providing guidance not just on what language to use to talk about people with a lived and living experience (Language statement 11. ‘Person‐first language should be used when describing someone's experience’), but in providing guidance on how they should be included in language decisions (Language statement 2. ‘When deciding the language to use, consulting with people who have personal and lived experience is important’; Language statement 10. ‘Where possible, the words used to describe a person's experience should be aligned with their preferences’). This study's findings on image use were a more dramatic expansion on existing guidance, which typically focused solely on high‐level recommendations about avoiding stigmatising or sensationalised imagery. New guidance was offered, for example on image diversity, informed consent of people with a lived and living experience for use of their image, as well as the style and origins of images (Image statement 26. ‘Images that depict the diverse population in Australia are preferred over internationally sourced stock images’).

The findings also appeared largely consistent with most of the existing guidelines compared above in providing minimal culturally specific guidance on image and language use. In most cases, the focus was on individual‐level rather than social or cultural‐level recommendations. New Zealand's guidelines for media reporting on people with mental health concerns were exceptional in this instance, providing culturally specific guidance regarding Māori and Pacific Peoples, explanations of cultural concepts and practical examples using Te reo Māori (Māori language) [12]. In comparison, only broad cultural‐level recommendations found consensus in this study (Language statement 4. ‘Different cultures may have different beliefs or understandings when communicating about these issues. The views of the particular group should be sought in determining the most appropriate language’; Language statement 5. ‘Terms often used to describe mental illness or suicide may not exist or translate easily for people from culturally and linguistically diverse backgrounds’). One image statement with reference to specific cultural groups achieved consensus (Image statement 29. ‘Care should be taken to avoid tokenistic use of specific symbols and imagery (e.g., Aboriginal or Torres Strait Islander flags, LGBTIQ+ flag)’). Language statements specific to Aboriginal and Torres Strait Islander Peoples were included in the original survey but did not achieve consensus across all three panels. This is unsurprising when 95% of experts did not identify as either an Aboriginal or Torres Strait Islander person, and may have felt unable to speak to culturally specific items outside of their own experience. As noted in the results section, these and other terminology specific statements required further consultation with relevant stakeholder groups post‐study.

Qualitative feedback provided insight where there were discrepancies in consensus between the panels, showing that in some cases experts (particularly those from the professional communicators panel) had deferred to the expertise of those with lived experience or to sector professionals seen to have more specialised expertise. This was evidenced not only in the lack of language statements referring to specific terminology but also language and image statements relating to specific mental health concerns such as body image and disordered eating or AOD concerns as well as culturally specific guidance. These findings reflected real world conditions where media and other professional communicators can be seen as translators or interpreters of information provided by the mental health, suicide prevention or AOD sectors or people with lived experience rather than as content experts in and of themselves. While this may indicate a need to rethink using a professional communicators panel, their inclusion in the survey was to provide additional end‐user perspectives and to ensure that the final guidance and resulting resources were fit for purpose and reflected the challenges faced by professional communicators when reporting on or sharing stories about suicide, mental health concerns or AOD concerns. Guidance that is not adopted or used by the end user will have little impact on improving language or image use in the Australian content.

### 4.1 Strengths and Limitations

In addition to the challenges discussed above, this study had several strengths and limitations. One of the key strengths of the study, and the Words and Images project more broadly, was the inclusion of people with a lived and living experience of mental health concerns, suicide or AOD concerns. Combined with lived experience inclusion across other aspects of the Words and Images project, including in advisory groups, consultation and the project team, this followed the broad research and program development principle of ‘nothing about us, without us’, and the efforts of the mental health, suicide prevention and AOD sectors to increase leadership, co‐production and participation of people with lived experience [35, 36]. The inclusion of a lived experience expert panel in this study explicitly recognised lived experience as a form of expertise, with equal weighting given to each panel throughout the study.

A limitation of the study was a lower‐than‐expected retention rate between survey rounds (62.5%, or 25 of 40 experts completing the second round). A drop‐off rate of 20% was initially expected over the two rounds, in accordance with previous Delphi studies [37, 38]. Possible explanations for this attrition rate were a long round 1 survey and consequent time commitment as well as the timing of the Delphi survey [39]. The panel recruitment and survey rounds were conducted in early to mid‐2022 and may indicate a level of ‘survey fatigue’ [40] as research pivoted to non‐contact methods as part of the COVID pandemic response in Australia, where extensive lockdowns were experienced in Melbourne and Sydney throughout 2020 and 2021. This low retention rate may have influenced the outcome of consensus on marginal items in the second round, including statements on culturally specific terminology or statements that required more specialised knowledge [39]. It should be noted, however, that the final round response size was over twice the lower limit threshold of 12 experts [41]. Twenty‐five experts completed the second‐round survey, with comparable Delphi studies showing stable results with 22 or 23 experts [38]. This indicates that while some marginal items may have been impacted, the general strength of the findings remained.

## 5 Conclusion

This study aimed to develop best‐practice guidance for the use of language and images related to mental health, mental health concerns, suicide and AOD concerns based on consensus between three expert groups. This national approach to communication provides an important opportunity to improve quality and consistency of language and image use across Australia, in the media and other sites of communication where people may be exposed to harmful or helpful words and images. Although existing guidelines were identified in the scoping of the wider project and have been shown to improve the quality of media reporting, opportunities for more nuanced guidance were also identified. Little was known, for example, about the types of images that are acceptable and preferred in relation to public communication about mental health, mental health concerns, suicide and AOD concerns. A lack of consistency in language use across policy and the mental health and suicide prevention sectors was also identified. A Delphi approach was used to achieve consensus amongst those considered experts where evidence and consistency were lacking. The findings help, in part, to encourage greater consistency in language and image use in Australia, but also highlight areas where consistency may not be possible or even warranted, such as in culturally specific settings.

Consensus ratings and qualitative feedback from 40 experts in two Delphi survey rounds provided clear, broad‐level guidance that formed the foundation for two new sets of guidelines. The first, *Images matter* (released in October 2022), focuses on safe and sensitive image use when communicating about mental health, mental health concerns, suicide and AOD concerns. The first of its kind, *Images matter* serves as an important first step towards improved understanding about depictions of these experiences using images, as well as a collective national effort to challenge stigmatising instances wherever they occur. These guidelines were launched as part of a suite of resources, which included a royalty‐free online image collection, with photographs developed as exemplars of the guidance emerging from this Delphi study. Alongside quick reference cards and a checklist, this suite of resources encourages the uptake of guidance across media and professional communicators embedded within the mental health, suicide prevention or AOD sectors.

The second set of guidelines, *Our words matter*, was released in April 2023 following further consultation with people with lived and living experience and further analysis of two large scale research projects by the University of New England and the University of Melbourne to further understand the nuance and guidance needed on language within the Australian context. The *Our words matter* guidelines consequently include sections on preferences and stigma around the use of diagnostic language, and the language of distress where a lack of shared language creates gaps in clinicians' perceptions of the level or severity of distress being experienced. *Our words matter* was also launched with a supporting suite of resources, including quick reference guides for researchers and service providers, quick reference language cards as well as a dynamic online glossary where specific terms were provided with a definition, usage guide, alternative language options and an indication of preferences amongst particular groups. Each of these resources acknowledges the importance and complexity of language, building on the guidance developed in this Delphi study.

Both resource suites are branded and hosted on the Mindframe website, alongside the Mindframe guidelines already used by the media and mental health, suicide prevention and AOD sectors in Australia, and the principles from *Images matter* and *Our words matter* integrated into ongoing training. It is hoped these new guidelines prove useful for Australian communities to communicate safely and sensitively, and with a more coherent voice about suicide, mental health concerns and AOD concerns. While they provide a new direction for guidance on language and images, it remains vital that we listen to people with lived and living experience, pay attention when language evolves and stay open to change.

## Funding

This research was funded by the National Mental Health Commission, Australia.

## Ethics Statement

Ethical approval for this study was provided by NSW Hunter New England Local Health District Research Ethics Committee (HNELHD‐HREC, 2022ETH00314).

## Conflicts of Interest

The Mindframe program is funded by the Australian Government Department of Health and Ageing through the National Suicide Prevention Leadership and Support Program. At the time of research, authors E.P., J.P., and J.S. were employed by Everymind, which administers the Mindframe Program.

## Supporting information


**Data S1:** Appendix A: Endorsed statements.

## Data Availability

Research data are not shared for privacy reasons, as some participants may be identifiable in qualitative data.
