# Supplementary material for: Developing Language and Image Use Guidance for Communicating About Suicide, Mental Health Concerns and Alcohol and Other Drug Concerns: An Australian Delphi Study
Source: Health Promot J Austr. 2026 Aug 14;37(4):e70236. doi: 10.1002/hpja.70236 (PMC13476173; doi:10.1002/hpja.70236)
Supplement: Supplementary file 1 [file HPJA-37-0-s001.docx]

**APPENDIX A: Endorsed statements**

**Language statements**

*General statements*

1. The language used in communication should be non-judgemental and non-stigmatising.
2. When deciding the language to use, consulting with people who have personal and lived experience is important.
3. Where possible, the words used to describe a specific community or population group’s experience should be aligned with their preferences.
4. Different cultures may have different beliefs or understandings when communicating about these issues. The views of the particular group should be sought in determining the most appropriate language.
5. Terms often used to describe mental illness or suicide may not exist or translate easily for people from culturally and linguistically diverse backgrounds.
6. Inclusive language that acknowledges and honours the diversity of people’s experiences, identities, and bodies can also help to heal past trauma for people of diverse sexualities and genders.
7. Language should promote the ability of individuals and communities to enact positive change.
8. Where possible, language that is being used to describe experiences (e.g., ‘mental illness’, ‘distress’) should be defined or explained so that the audience is aware of the term’s meaning and why it is being used.
9. The term ‘commit’ should not be used in general communication about suicide as the term’s association with crime and/or sin can be stigmatising.

*Language used to describe personal or lived experience*

10. Where possible, the words used to describe a person’s experience should be aligned with their preferences.
11. Person-first language should be used when describing someone’s experiences (e.g., ‘a person experiencing’ or ‘a person living with’).
12. Avoid words that suggest a lack of quality of life for people (e.g., a ‘victim’ of, or as ‘suffering from’).
13. Combat related language and terms such as ‘war on drugs’ or ‘combat suicide’ should be avoided. General terms (e.g., ‘address’, ‘reduce’, ‘support’) are preferred.

*Suicide and self-harm*

14. The term ‘commit’ should not be used when communicating about suicide as it has been associated with crime and/or sin.
15. Language should not present suicide as a desired outcome (e.g., ‘successful suicide’ or ‘unsuccessful attempt’).
16. The word suicide should not be used out of context (e.g., ‘political suicide’).
17. Self-harm should never be described as manipulative or attention seeking.
18. Do not label people who have harmed themselves (e.g., ‘cutters’, ‘attempters’).
19. Do not label people who have attempted or died by suicide by the method used (e.g., ‘jumpers’).
20. Avoid language that broadly relates to methods when discussing suicide or self-harm (e.g., ‘roped in’, ‘trigger-happy’).
21. Avoid giving details about the method or location of a suicide attempt or death.
22. Avoid using language that suggests suicide is a “solution” to problems or life stressors.
23. Avoid using language that suggests suicide is a positive outcome (e.g., ’relief’, ‘finally at peace’).

*Alcohol and other drugs*

24. Outdated and derogatory terms associated with alcohol and other drugs used should not be used (e.g., ‘junkie’, ‘user’, ‘addict’).
25. Use accurate plain language to describe substance use (e.g., ‘problematic substance use’) rather than colloquial terms (e.g., ‘drug habit’).

*Mental illness*

26. Outdated and derogatory terms associated with mental illness should not be used (e.g., ‘psycho’, ‘nutter’).
27. Use accurate terms to describe treatments for mental illness and refrain from using outdated and derogatory terms (e.g., ‘happy pills’, ‘psych ward’).
28. Language used to describe mental health services should not imply that they are similar to prisons (e.g., ‘discharged’ rather than ‘released’ from hospital).
29. Diagnostic terms should not be used out of context (e.g., ‘bipolar weather’).

*Medical and research terms*

30. When presenting data and statistics, sensationalist language (e.g., ‘skyrocket’ or ‘epidemic’) should not be used.
31. Some data and research terms are stigmatising when used in public communication (e.g., ‘burden’, ‘deliberate self-harm’, ‘copycat’). Care should be taken to use plain English to describe outcomes of research rather than terms that can cause offence.
32. Care needs to be taken when using health service terms that are potentially disempowering (e.g., ‘assertive outreach’) or judgemental (e.g., ‘treatment resistant’).
33. The term ‘mental health’ should be used to describe a positive concept of wellbeing, not merely the absence of mental illness.
34. Public communication should use simple, everyday language, rather than medical or clinical terminology, unless the medical or clinical terminology is widely understood.

*Terminology*

35. Consistent use of language relating to mental illness and suicide may help to increase understanding and decrease stigma in Australia. It is preferable that the same or similar terms are used across different sectors and communities when communicating about these issues.

*New statements*

36. Using simple English to describe mental illness or suicide would increase accessibility.

**Image statements**

*General statements*

1. Images should include a diversity of ages, genders, cultures and geographies.
2. Images should include a diversity of relationships.
3. Images should include a diverse range of body shapes and sizes, especially when communicating about body image and/or eating disorders.
4. Images that show people in the company of others are preferred over images that show people alone.
5. Images that show people accessing support from relevant and available services can be helpful.
6. Images that show people accessing support from friends, family or other social connections can be helpful.
7. Images should try to balance promoting hope with showing the reality of living through challenging experiences.
8. Images should depict people’s journeys and experiences as a process rather than an end point.

*Images of people with personal or lived experience*

9. Images of people who have personal or lived experience should be used with their knowledge and permission.
10. People with personal or lived experience who provide their image for use should be able to determine how it is used (e.g., types of communication, length of time).
11. People with personal or lived experience should be able to withdraw permission for use of their image.
12. Care needs to be taken when using images of people who have personal or lived experience as these can impact their lives, now and into the future.

*Perpetuating stereotypes and stigma*

13. Images can be used to improve understanding of mental health and wellbeing, including people’s individual experiences.
14. Images that include stigmatising words in the graphics (e.g., psycho) should not be used.
15. Images should avoid depicting a power imbalance between people providing support and people receiving support.
16. Images should not stigmatise treatment and support or specific types of support.
17. Images should not sexualise or objectify the issues or individuals depicted.

*Suicide and self-harm*

18. Images should not show methods of suicide in any way.
19. Images should not include illustrations, diagrams or photos of implements that may be used in self-harm or suicide.
20. Images should not use design elements or patterns which could indicate a method of suicide (e.g., patterns that look like rope).
21. Images should not show specific locations where suicides have occurred.
22. Images that show problematic websites or places where people can access details about methods of suicide should be avoided.
23. Care should be taken when using images of self-harm (e.g. cuts on the body; scars) as, in some contexts, these may encourage imitative behaviour.
24. In general communication about suicide, images associated with a method of suicide should be avoided (e.g. tree branch, rope, cliff edge).
25. In general communication about suicide, images should not show locations where suicides could occur (e.g. cliff edges, bridges, tall buildings).

*Alcohol and other drugs*

NA

*Body image and eating disorders*

NA

*Style and types of images used*

26. Images that depict the diverse population in Australia are preferred over internationally sources stock images.
27. Repeated use of the same stock images to communicate about mental health issues should be avoided.
28. Image use should align with international standards for accessibility (e.g., including text that adequately describes the image for text readers).
29. Care should be taken to avoid tokenistic use of specific symbols and imagery (e.g., Aboriginal or Torres Strait Islander flags, LGBTIQ+ flag).
30. Images that use illustrations or cartoons can be used, as long as they are not stereotypical or stigmatising.

*New statements*

31. Images used to depict mental health treatment or services should be Australian-specific and avoid outdated or harmful stereotypes (e.g., white coats and stethoscopes; straightjackets).
32. People who have personal or lived experience should be provided with clear information about the use of their images including the risks and benefits of their participation.
33. Care should be taken in any public communication where mental illness is being linked to violence (e.g., in reporting on a court proceeding) in order to avoid increasing stigma for others in the community with the same diagnosis.
34. Care should be taken when using images of memorials in public communication, as these may show specific locations where suicides have occurred.
